# Altitude-Dependent Morphophysiological, Anatomical, and Metabolomic Adaptations in *Rhodiola linearifolia* Boriss.

**DOI:** 10.3390/plants13192698

**Published:** 2024-09-26

**Authors:** Nina V. Terletskaya, Malika Erbay, Aigerim Mamirova, Kazhybek Ashimuly, Nazym K. Korbozova, Aigerim N. Zorbekova, Nataliya O. Kudrina, Matthias H. Hoffmann

**Affiliations:** 1 Faculty of Biology and Biotechnology, Al-Farabi Kazakh National University, Al-Farabi 71, Almaty 050040, Kazakhstan; malika.isa99@mail.ru (M.E.); kajeke@mail.ru (K.A.); naz-ik@mail.ru (N.K.K.); zorbekova92@mail.ru (A.N.Z.); kudrina_nat@mail.ru (N.O.K.); 2 Institute of Genetic and Physiology, Al-Farabi 93, Almaty 050040, Kazakhstan; 3 Wittenberg Institut für Geobotanik und Botanischer Garten, Martin-Luther-Universität Halle, Am Kirchtor 3, D-06108 Halle, Germany; matthias.hoffmann@botanik.uni-halle.de

**Keywords:** *R. linearifolia* Boriss., mountains, altitude, morphology, anatomy, pigments, metabolome

## Abstract

*Rhodiola linearifolia* Boriss., a perennial alpine plant from the *Crassulaceae* family, is renowned for its unique medicinal properties. However, existing research on this species is limited, particularly regarding the impact of altitude on its physiological and medicinal compounds. The current study employed morphophysiological and anatomical methods to explore the adaptive mechanisms of *R. linearifolia* across different altitudinal gradients, while also examining photosynthetic pigments and metabolomic changes. Our results indicate that despite the simultaneous effects of various mountain abiotic factors, significant correlations can be identified between altitude and trait variation. An optimal growth altitude of 2687 m above sea level was identified, which is pivotal for sustainable ecosystem management and potential species introduction strategies. It is noted that increasing altitude stress enhances the synthesis of secondary antioxidant metabolites in *R. linearifolia*, enhancing its pharmaceutical potential.

## 1. Introduction

Plants growing at high altitudes face a variety of abiotic stresses daily, including fluctuations in temperature, humidity, solar radiation, and atmospheric pressure [1,2]. These environmental challenges significantly influence their growth and development. High-mountain plants have evolved a range of physiological and chemical strategies to cope with high-stress environments. Species adapted to large altitudinal gradients develop complex, multifaceted adaptive features to survive [3].

Generally, high-altitude plants are understudied yet possess significant phytochemical potential due to their unique metabolic adaptations, making them promising candidates for pharmaceutical applications. Extensive research indicates that the synthesis and accumulation of biologically active compounds in these plants are intimately linked to their environmental conditions [4,5,6,7]. Additionally, elevation-dependent natural climate gradients offer valuable insights for biologists studying plant responses to climate change. Such studies are crucial for predicting phenotypic adaptations, phenological synchronizations, and secondary metabolite biosynthesis, which are key survival strategies in evolving ecosystems [2,3,8,9].

The Shymbulak tract is situated on the northern slope of the Ile Alatau range, part of the Ile-Alatau State National Park which spans over 200,000 hectares, at elevations ranging from 2200 to 3200 m above sea level (m a.s.l.). It falls within the Zailiyskiy mountain subprovince of the Dzungar–Northern Tien Shan province, in the Iran–Turan subregion and the Sahara–Gobi Desert region, according to the latest botanical and geographical zoning of Kazakhstan and Central Asia [10]. The climate of the area is characterized by its continental highland nature, featuring sharp temperature fluctuations both daily and annually. Seasonal influences include warm subtropical air masses from Iran and Central Asia, which can cause brief winter warm-ups. Conversely, the intrusion of cold air from Siberia brings clear, frosty conditions. Atlantic winds introduce cloudy weather with heavy snowfalls in winter and showers in summer. The region receives an average of 2467 h of sunshine annually, with a significant increase from winter (124–140 h per month) to summer (285–316 h per month). Total annual solar radiation under clear skies reaches 7439 MJ m^−2^, with winter direct solar radiation ranging from 266 to 391 MJ m^−2^ and summer values rising to 824–958 MJ m^−2^. The average annual air temperature at Shymbulak is 4.4 °C, peaking at 14.9 °C in July and dropping to −6.0 °C in January, with an annual temperature range of 20.9 °C. The frost-free period averages 104 days. The average annual temperature reaches negative values at elevations above 2700 m a.s.l. Annual precipitation totals 935 mm, with 693 mm falling between April and October, accounting for 74% of the yearly total (http://ecodata.kz:3838/dm_climat_ru/). The study area, located within the subalpine-type juniper-steppe-meadow belt at 2500–3000 m a.s.l., is noted for its remarkable floristic and phytocoenosis diversity. The vegetation predominantly consists of medium-grass, cryophyte, subalpine-type meadows dominated by cereals such as *Dactylis glomerata*, *Alopecurus soongoricus*, *Avenastrum pubescens*, *Poa sibirica*, *Hierochloe odorata*, and *Agropyron ugamicum*. The area is also rich in forbs including *Rhodiola linearifolia*, *Doronicum turkestanicum*, *Pyrethrum karelinii*, *Phlomis oreophila*, *Trollius dschungaricus*, *Delphinium confusum*, and *Aquilegia glandulosa*. Extensive juniper thickets (*Juniperus pseudosabina* and *J. sabina*) with a creeping form cover large areas. The soils are primarily mountain-meadow subalpine and high-mountain meadow-steppe leached types. While the vegetation of the Ile Alatau is generally well documented, the specific area around the Shymbulak Mountain Resort remains underexplored in botanical studies.

*Rhodiola linearifolia* Boriss., a perennial high-mountain plant, grows from the upper forest line, on rocky terrains up to 3000 m a.s.l. *R. linearifolia* is native to the northeastern Tianshan mountain system. In some literature, it is occasionally merged with *R. kirilowii*, which has a broader distribution, extending from the western Tianshan across the Pamir Mountains to the Himalayas. Notably, *R. kirilowii* occupies higher altitudes within the Tianshan region [11]. This species is of particular interest due to the renowned medicinal properties of its family, *Crassulaceae*, notably the well-known yet endangered *Rhodiola rosea* L. Botanically related plants often share similar chemical compositions, suggesting that other *Rhodiola* species could exhibit comparable pharmacological effects. *R. rosea*, for instance, is celebrated for its stimulating and adaptogenic properties [12]. Kyrgyz scientists have observed significant stress-protective effects from a liquid extract of *R. linearifolia*, particularly in models of acute stress exposure. This extract normalized peripheral blood and biochemical indices affected by stress [13]. Despite these findings, comprehensive studies on *R. linearifolia* remain scarce, particularly regarding how altitude affects its physiological and medicinal properties. In addition, we believe it is crucial to study the properties of the above-ground parts of the plant without damaging the root system, to prevent the loss of the species, as occurred with *Rhodiola rosea* L. The current study utilized morphophysiological and anatomical methods to explore the adaptive mechanisms of *R. linearifolia* across different altitude gradients. A quantitative and qualitative assessment of leaf pigments served as a sensitive indicator of the plant’s physiological state and photosynthetic apparatus, highlighting directional adaptive responses to stress factors.

We assume that as altitude increases, the combination of stress factors affecting plants also intensifies. Consequently, high-altitude plants must employ various adaptation mechanisms for their growth and development, with the efficiency of these mechanisms likely correlating with the altitude at which the plants grow. Understanding the clear link between the physiological adaptation mechanisms of the high-mountain medicinal species *R. linearifolia* and changes in its metabolome will allow us to identify biologically active substances that not only support the plant’s survival in extreme highland conditions but may also possess valuable properties for human use.

The examination of plant metabolome variations at varying altitudes provided insights into the physiological, biochemical, phenotypic, and morphological responses of plants to environmental changes [6,7,14,15]. The application of GC-MS metabolomics has been particularly effective in analyzing plant metabolic adaptations under stress conditions [16].

The findings obtained in the current research are critical not only for understanding how morphophysiological mechanisms and secondary metabolites assist plants in coping with abiotic stress in high-altitude ecosystems, but they also have significant implications for pharmacology, sustainable ecosystem management, and the potential introduction of valuable species.

## 2. Results

### 2.1. Morphometric Parameters

**Figure 1:**
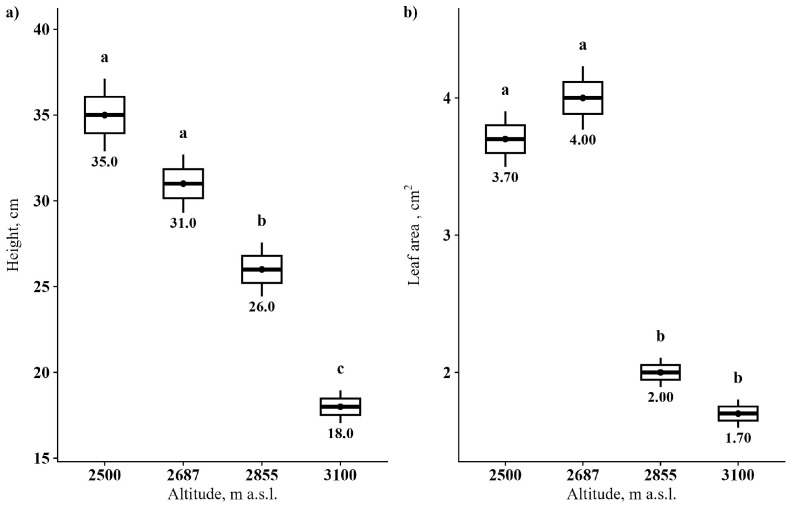
*R. linearifolia* morphometric parameter alterations depending on altitude. Note: (**a**) plant height, cm; (**b**) leaf area, cm^2^. Different letters within one parameter show significant difference (*p* < 0.05).

Figure 1 illustrates that the height and leaf area of *R. linearifolia* plants significantly decreased with increasing altitude. Notably, there was a slight increase in leaf area at 2687 m a.s.l. compared to the lowest altitude.

### 2.2. Anatomical Parameters

**Figure 2:**
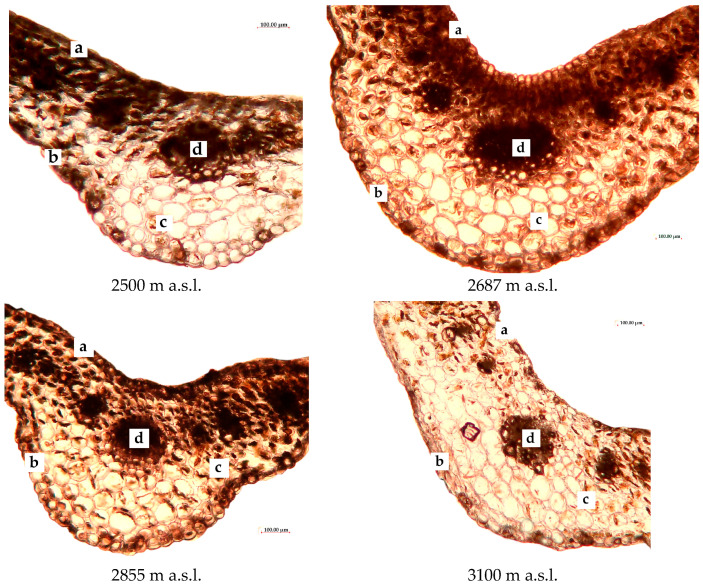
Anatomical structure of *R. linearifolia* leaves depending on altitude. Note: a—adaxial epidermis; b—abaxial epidermis; c—mesophyll; d—central vascular bundle. Scale bar = 100 µm.

Figure 2 demonstrates the anatomical structure of *R. linearifolia* leaves.

Changes in leaf size and their anatomical components are visually apparent. According to Table 1, almost all measured anatomical leaf parameters generally decreased with increasing altitude. Notably, the thicknesses of both the abaxial and adaxial epidermis significantly decreased at the higher altitudes (2855 and 3100 m a.s.l.) compared to the lowest elevation measured.

At an altitude of 2687 m a.s.l., there was a notable increase in the thickness of the central vein and the diameter of the central vascular bundle of the *R. linearifolia* leaf compared to the lowest latitude, surpassing values observed at 2855 and 3100 m a.s.l. However, the lowest values for the diameter of the central vascular bundle and the thickness of the mesophyll were recorded at 2855 m a.s.l.

**Figure 3:**
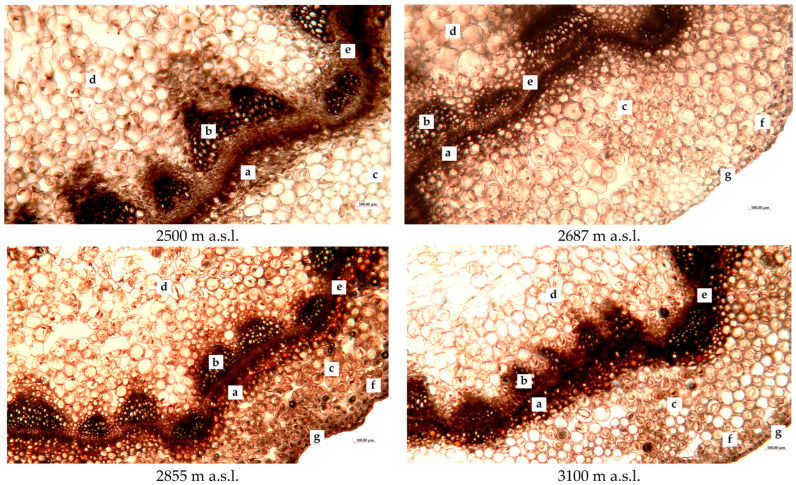
Anatomical structure of *R. linearifolia* stems depending on altitude. Note: a—phloem; b—xylem; c—chlorenchyma; d—parenchyma; e—sclerenchyma; f—collenchyma; g—epidermis. Scale bar = 100 µm.

Figure 3 illustrates the anatomical parameters of the *R. linearifolia* stems.

Similar to leaf sections, stem sections of *R. linearifolia* showed visually discernible changes in anatomical structures, including a reduction in vascular bundles. Notably, red inclusions were observed in the sclerenchyma of stems collected at higher altitudes (2855 and 3100 m a.s.l.), which are visible in unstained preparations. This may suggest an increased accumulation of secondary metabolites.

According to Table 2, all measured stem anatomical parameters also tended to decrease with increasing altitude. However, at 2687 m a.s.l., certain parameters such as stem collenchyma, chlorenchyma, and parenchyma thicknesses were significantly higher compared to the lowest altitude, surpassing values at 2855 and 3100 m a.s.l. Conversely, chlorenchyma thickness, vascular bundle diameter, and parenchyma thickness were the lowest at 2855 m a.s.l.

**Figure 4:**
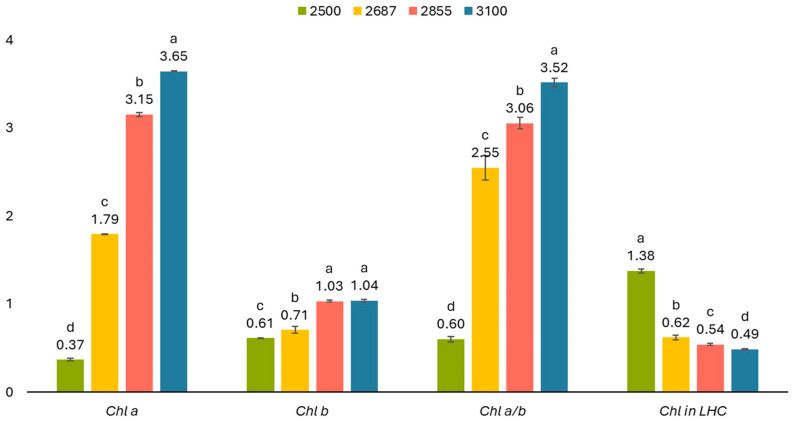
Chlorophyll pigments content (mg g^−1^ FW) in *R. linearifolia* shoots depending on altitude. The bars are arranged with increasing altitude from left to right. Different letters within one parameter show significant difference (*p* < 0.05).

To evaluate our hypothesis on the influence of altitude on photosynthetic parameters, the content of photosynthetic pigments, including chlorophyll *a*, chlorophyll *b*, total chlorophylls (*a* + *b*), the chlorophyll *a*/*b* ratio, and chlorophyll content in light-harvesting complexes in *R. linearifolia* shoots at various altitudes, was measured (Figure 4). The content of chlorophylls *a* and *b* in the shoots increased with increasing altitude. However, while the chlorophyll *a*/*b* ratio also rose, the total chlorophyll content in the light-harvesting complexes significantly decreased (Figure 4).

**Figure 5:**
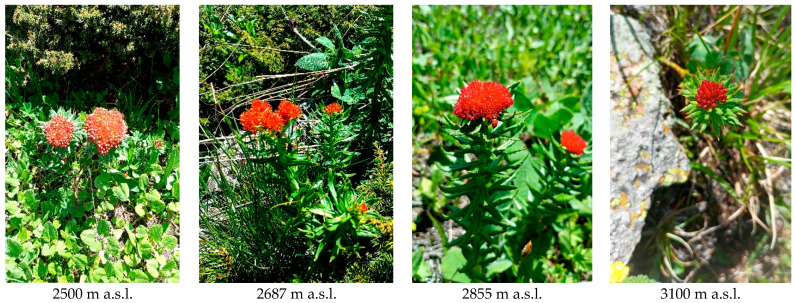
*R. linearifolia* flower color alterations depending on altitude.

Carotenoids play a crucial role not only in plant photosynthesis but also in coloring petals and fruits. Observing an intensification of flower color with increasing altitude in *R. linearifolia* (Figure 5), the total carotenoid content in the flowers and shoots of plants at various altitudes was analyzed (Figure 4).

**Figure 6:**
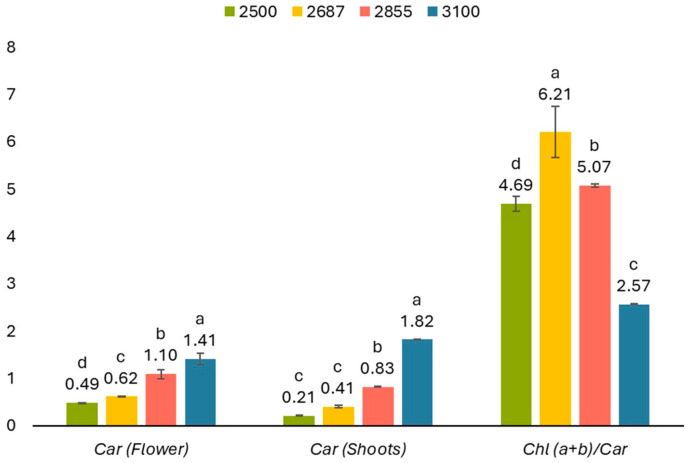
Changes in carotenoid content in flowers and shoots of *R. linearifolia* depending on altitude. The bars are arranged with increasing altitude from left to right. Different letters within one parameter show significant difference (*p* < 0.05).

Carotenoid content in *R. linearifolia* flowers and shoots showed a direct increase with altitude, rising from 0.49 ± 0.001 to 1.41 ± 0.12 mg g^−1^ FW and from 0.21 ± 0.01 to 1.82 ± 0.02 mg g^−1^ FW, respectively (Figure 4 and Figure 6). Additionally, the ratio of chlorophyll *a* + *b* to carotenoids displayed a notable peak at 2687 m a.s.l., followed by a decrease at higher altitudes.

### 2.3. Metabolome Alterations

**Figure 7:**
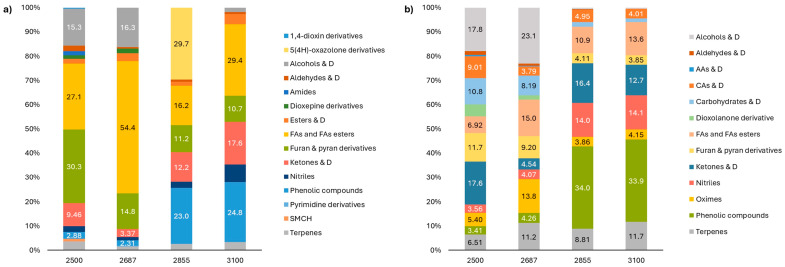
Metabolome alterations in flowers (**a**) and shoots (**b**) of *R. linearifolia* depending on altitude. Note: AA—amino acid; CA—carboxylic acid; D—derivatives; FA—fatty acid; SMCH—saturated monocyclic hydrocarbons.

Results of metabolic analysis revealed significant changes in *R. linearifolia* plants depending on altitude (Figure 7, Tables S1 and S2)). Notably, an increase in the concentration of phenolic compounds in both the flowers and shoots was observed as the altitude increased.

With increasing altitude, the flowers of *R. linearifolia* exhibited a tendency to increase the content of nitriles, oximes, ketones, and their derivatives, while a tendency to decrease in furan and pyran derivatives was observed (Table S1). 

In the shoots, there was a notable rise in nitriles, along with a tendency toward an increase in fatty acids, fatty acid esters, and terpenes. Conversely, there was a decrease in the concentration of carbohydrates and their derivatives, aldehydes and their derivatives, amino acids and their derivatives, dioxolanone derivatives, as well as furan and pyran derivatives (Table S2)

**Figure 8:**
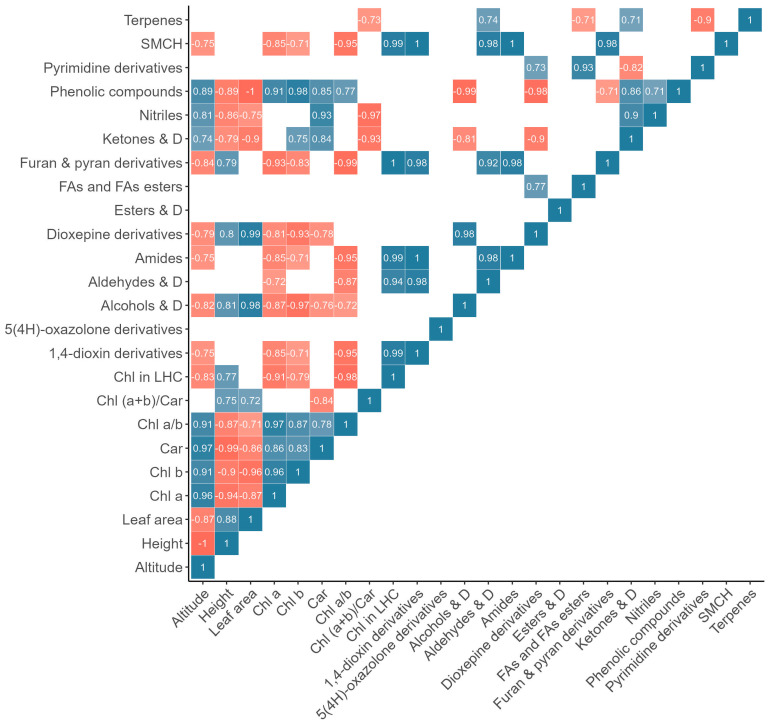
Pearson correlation heatmap (for the metabolite content in flowers). Note: Car—carotenoids; LHC—light-harvesting complex; D—derivatives; FA—fatty acid; SMCH—saturated monocyclic hydrocarbons.

**Figure 9:**
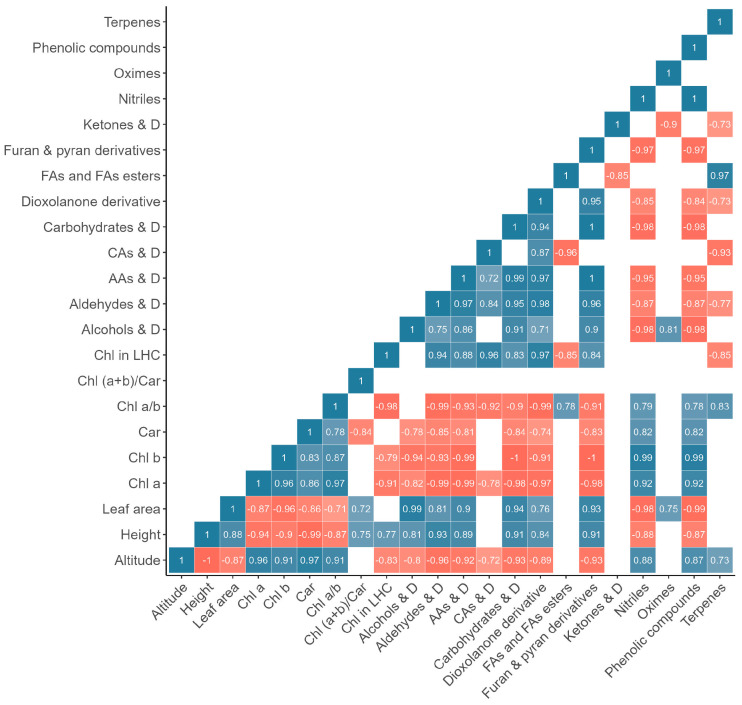
Pearson correlation heatmap (for the metabolite content in shoots). Note: Car—carotenoids; LHC—light-harvesting complex; D—derivatives; AA—amino acid; CA—carboxylic acid; FA—fatty acid.

Altitude correlated positively with the levels of chlorophyll *a*, *b*, carotenoids, and the chlorophyll *a* to *b* ratio (Figure 8 and Figure 9). Conversely, plant height (−1), leaf area (−0.87), and chlorophyll content in LHC (−0.83) negatively correlated with altitude. 

Considering the metabolite content in plant flowers, a sufficiently strong negative correlation was observed between altitude and six metabolites, specifically furan and pyran derivatives (−0.84), alcohols and their derivatives (−0.82), dioxepine derivatives (−0.79), 1,4-dioxin derivatives (−0.75), amides (−0.75), and saturated monocyclic hydrocarbons (−0.75). Furthermore, phenolic compounds (0.89), nitriles (0.81), and ketones and their derivatives (0.74) positively correlated with altitude (Figure 8).

In the case of metabolite content in plant shoots, the content of furan and pyran derivatives (−0.93), aldehydes and their derivatives (−0.96), carbohydrates and their derivatives (−0.93), amino acids and their derivatives (−0.92), dioxolanone derivatives (−0.89), alcohols and their derivatives (−0.80), and carboxylic acids and their derivatives (−0.72) negatively correlated with altitude (Figure 9). Nitriles (0.88), phenolic compounds (0.87), and terpenes (0.73) content positively correlated with altitude. The content of fatty acids and fatty acid esters, ketones and their derivatives, and oximes showed no correlation with altitude (Figure 9).

Among the eight groups of metabolites identified in both flowers and shoots of *R. linearifolia*, (i) fatty acids and fatty acid esters showed no correlation with altitude in either flowers or shoots; (ii) alcohols, their derivatives, and furan and pyran derivatives exhibited a negative correlation with altitude in both flowers and shoots; (iii) nitriles and phenolic compounds positively correlated with altitude in both flowers and shoots; (iv) in flowers, aldehydes, their derivatives, and terpenes showed no correlation with altitude, while in shoots, aldehydes and their derivatives correlated negatively, and terpenes correlated positively with altitude; (v) ketones positively correlated with altitude in flowers, but no correlation was observed in shoots.

## 3. Discussion

It is well documented that challenging conditions along elevation gradients, such as increasing soil pH and decreasing temperature coupled with increasing solar radiation, significantly influence plant growth [9,17]. These factors contribute to a notable negative correlation between altitude and morphological features, predominantly impacting plant traits and resource allocation in high-mountain regions [18]. Typically, phenotypic plasticity and physiological adaptations result in decreased plant height and leaf area with increasing altitude [19,20], aligning with our experimental data. Plants adapt by slowing growth and allocating more resources to defensive structures to enhance survival in environments with increased radiation and lower temperatures [21]. For instance, a reduced leaf area diminishes solar energy absorption and transpiration, thereby mitigating leaf damage from intense UV radiation and strong winds [22]. These adaptations necessitate higher energy expenditure in forming denser tissue layers for structural protection [23]. However, our findings reveal that these morphometric and anatomical changes do not strictly follow a linear trend with altitude increases. Interestingly, a slight increase in leaf area and certain anatomical parameters at 2687 m a.s.l. suggests this elevation may be more conducive for *R. linearifolia* growth compared to our initial reference point of 2500 m a.s.l. In contrast, the stress effects on plants were more pronounced at 2855 m a.s.l., underscoring that the optimal conditions for *Rhodiola* populations are above 2500 m a.s.l. but below 2855 m a.s.l., and closer to 2687 m a.s.l.

The photosynthetic apparatus is fundamentally composed of light-harvesting pigment-protein complexes within the photosynthetic membranes. These complexes convert sunlight into electron excitation energy, which is then transferred to the photosystems’ reaction centers for primary energy storage [24,25]. The content of photosynthetic pigments, such as chlorophylls and carotenoids, is crucial for assessing plant viability, environmental response, and resistance to abiotic stresses. These pigments not only influence biomass yield but are also indicative of species-specific traits [26,27]. Given their role in light absorption and photochemical reactions in chloroplasts, analyzing the pigment pool of the photosynthetic apparatus is essential for understanding the dynamics of photosynthesis.

In particular, the concentration of chlorophyll is crucial for photosynthesis and the biological productivity of plants, and parameters such as the chlorophyll *a*/*b* ratio are key indicators of a plant’s adaptability to environmental changes [28,29]. In our study, an increase in altitude corresponded with a significant rise in both total chlorophylls and the chlorophyll/carotenoids ratio, aligning with the literature [30]. The chlorophyll *a*/*b* ratio typically ranges from 2 to 3.5 in many terrestrial plant species [31,32], reflecting findings from our experiments except at the lowest altitude, where *R. linearifolia* showed suboptimal ratios. Chlorophyll *a* dominates the total chlorophyll pool, while chlorophyll *b*, which functions protectively and screens the photosynthetically active chlorophyll *a*, is more susceptible to stress damage and can be converted into chlorophyll *a*, resulting in an observed increase in chlorophyll *a* content under stress [33,34,35,36]. Therefore, the observed increase in the chlorophyll *a*/*b* ratio with altitude might serve as a protective mechanism, potentially reducing damage from reactive oxygen species (ROS) [37].

With increasing altitude, starting from 2687 m a.s.l., the proportion of chlorophylls in the light-harvesting complexes (LHC) of *R. linearifolia* significantly decreased. This observation suggests that high-altitude conditions induce notable structural changes in the photosynthetic machinery, possibly due to accelerated degradation of chlorophyll under stress or disruptions in its biosynthesis [34,38]. This degradation likely results from damage to chloroplast membranes and structures, heightened chlorophyllase activity, and chlorophyll photooxidation [39].

Carotenoids, critical lipid compounds with unsaturated double bonds, enhance the flexibility of chloroplast membranes, thereby stabilizing the photosynthetic apparatus under stress [32,40]. As auxiliary pigments, carotenoids efficiently transfer absorbed energy to chlorophyll (15–90% efficiency) and shield it from excessive light intensity, preventing the photooxidation of protoplasmic organic compounds in the presence of free oxygen [26,40,41].

Although limited information exists on the relationship between carotenoid variations and altitude adaptation in plants, significant correlations have been observed. For example, a study on Tibetan peach fruit found that carotenoid levels and diversity, which are substantial at high altitudes, play a crucial role in plant adaptation to such conditions [42]. Typically, carotenoid content in land plant leaves varies from 0.2 to 5.1 mg g^−1^ and closely correlates with chlorophyll levels [32]. In our study, a strong positive correlation between carotenoids and chlorophylls *a* and *b* was noted (Figure 8). We observed a marked decrease in the chlorophyll-to-carotenoid ratio (*Chl* (*a* + *b*)/*Car*) starting from the altitude of 2687 m a.s.l., alongside an increase in carotenoid levels. This suggests a misalignment between light absorption and photosynthetic activity [43], indicating that the increased carotenoid content may serve a protective function by dissipating excess energy and scavenging free radicals.

The literature suggests that accelerated carotenoid accumulation may lead to a transition of chloroplasts into chromoplasts, affecting photosynthetic efficiency [44,45,46]. This transition is particularly relevant in floral chromatics, where carotenoids like α-carotene and β-carotene enrich color intensity and diversity—a response likely modulated by specific gene expressions influenced by environmental factors such as solar radiation and temperature changes [47].

Plants continually navigate a trade-off between growth and defense, particularly in their primary and secondary metabolism [9,48]. This balance is guided by the optimal defense theory, which posits that metabolomic changes ensure plants strategically allocate resources between growth and defense based on their developmental needs and environmental pressures [49,50]. In high-altitude environments, secondary metabolites play a crucial role in plant adaptation. These metabolites help plants cope with stress factors typical of mountain ecosystems, such as low temperatures, enhanced UV radiation, and ROS presence [3,51].

In our study, the variation in the concentration of specific substances in plants growing at different altitudes was often nonlinear, reflecting the complex impact of multiple abiotic factors prevalent in mountainous environments. This complexity also extends to internal plant dynamics, particularly the donor–acceptor interactions between shoots and flowers, which challenges the strict application of the laboratory “principle of a single difference”. Despite these complexities, it is possible to discern general trends in the changes in substance concentrations and to attempt explanations for the observed patterns. Notably, during this stage of vegetation, the metabolomic spectrum of both shoots and flowers is markedly diverse and rich, both quantitatively and qualitatively.

Oxidative stress experienced by high-altitude plants is found to stimulate antioxidant enzymatic metabolism, crucially allowing chloroplasts to maintain low levels of lipid peroxidation. This adaptation involves fatty acids (FAs), synthesized de novo only in plastids, which play a vital role in protecting against various stress factors [52,53]. Predominantly, the mass spectra revealed that saturated palmitic acids (C16:0) and unsaturated FAs such as oleic (C18:1), linoleic (C18:2), and linolenic (C18:3) acids were detected not only in free forms in flowers but also as esters in both flowers and shoots.

FAs are integral to various biological functions: they are components of cell membranes in glycolipids, serve as carbon and energy reserves in triacylglycerol, act as extracellular barrier components, and function as precursors for bioactive molecules and stress signal regulators [53,54,55]. Moreover, the proportion of unsaturated FAs in the plasma membrane has been shown to correlate with membrane fluidity, which is pivotal for maintaining cellular function under stress [56]. Particularly under stress, an increased content of unsaturated FAs in the inner membranes of chloroplasts and mitochondria can alleviate photoinhibition of PSII [57,58]. Despite the absence of significant correlations between fatty acids and their derivatives and altitude in our observations, FAs and their derivatives constituted a significant portion of the metabolites in flowers (16.4–54.4%) and were less prevalent in shoots (6.92–15.0%), with the highest levels detected in plants at an altitude of 2687 m a.s.l.

Lipid composition in high-altitude plants is diverse, encompassing a range of components such as hydrocarbons, esters, aliphatic aldehydes, primary and secondary alcohols, (1.2-, 2.3-, α-, and ω-) diols, ketones, β-diketones, and triacylglycerols. Notably, alcohols and their derivatives dominate at lower altitudes (17.8–24.1%), but their concentration diminishes significantly as altitude increases. Furthermore, there is a discernible decrease in the levels of aldehydes in both flowers and shoots with increasing altitude. This aligns with findings by Kumari et al. (2020) [15], who reported that carbon source–sink partitioning, the tricarboxylic acid (TCA) cycle, ascorbate metabolism, and other metabolic pathways play crucial roles in plant adaptation to alpine environments. Our findings also show a consistent decrease in the concentration of saturated monocyclic hydrocarbons in flowers and shoots alongside an increase in altitude, accompanied by a reduction in carboxylic acids and their derivatives and carbohydrates in shoots. These changes may indirectly reflect disruptions in photosynthesis as altitude increases. Moreover, the levels of furan and pyran derivatives, which contain active alcohol, aldehyde, and ketone groups and are known for their high biological activity [59], also declined in both flowers and shoots of *R. linearifolia* with rising altitude.

In the case of ketones, we observed distinct patterns in their distribution in *R. linearifolia*, where ketone levels increased in flowers but decreased in shoots with elevation gain. This distribution is likely due to the dynamic regulation of metabolism, which optimally directs metabolites—essential building blocks—to developing organs and tissues [60]. Such changes suggest that ketones and their derivatives may enhance the stress resilience of *R. linearifolia* flowers under the influence of complex abiotic factors characteristic of high-altitude environments.

Additionally, terpenes, as well as other volatile components (aldehydes, alcohols, and esters) found in green leaves, serve as plant-to-plant stress signals [61]. Diterpene hydrocarbons, like phytols that form part of chlorophyll, are intricately linked to the *R. linearifolia* stress response when photosynthetic activity significantly changes [5]. Research suggests that volatile terpenes can mitigate oxidative stress effects either by direct intercellular reactions with oxidants or by modifying ROS signaling pathways [53,62]. The observed increase in terpene accumulation in shoots at higher altitudes, showing a high correlation relationship with elevation gain, even if this increase does not have a strict linear relationship, highlights the complex and varied nature of stress factors in mountainous regions, which can fluctuate even within short intervals.

5(4H)-Oxazolones, or azlactones, which were detected in *R. linearifolia* flowers at 2855 m a.s.l., serve as precursors for synthesizing a variety of biologically active substances, including oxoacids, amino acids, and diverse carbocycles and heterocycles [63,64]. Their presence is indicative of complex, ongoing transformations within the plant’s metabolome, responsive to environmental changes. This aligns with broader observations in plant biochemistry, where altitude is known to influence the accumulation of phenolic compounds, which in turn correlates positively with enhanced photoprotection and radical scavenging capabilities at higher elevations [65,66]. Phenols, capable of absorbing UV radiation across the range of 210 to 350 nm, play a critical role in modulating plant antioxidant defenses by mitigating ROS accumulation, thus enhancing stress tolerance at high altitudes [67,68,69]. While Hashim et al. [69] suggest that an increase in phenolic content could be due to a reduction in the activity of antioxidant enzymes, our current study did not measure these enzyme levels. However, our prior research indicates that plants begin to activate antioxidant enzymes as an early response to abiotic stress, well before any morphophysiological changes become apparent. It is also noted that severe stress, causing a transition from eustress to distress, might suppress antioxidant enzyme activities, suggesting a complex interplay between enzymatic and non-enzymatic responses to environmental stressors [70]. 

Rhodiola is recognized as a cyanogenic species, which means it can release hydrogen cyanide from damaged tissues—a mechanism that is part of a two-component chemical defense system in plants. Despite this, research suggests that the antioxidant activity of plants does not directly correlate with the presence of cyanogenic derivatives [71]. Over evolutionary time, cyanogenic glycosides have been shown to enhance plant plasticity, contributing to survival, viability, and resistance under environmental stresses. These compounds can transform into oximes, which may act as volatile protective agents, and further dehydration of these oximes can lead to hydroxynitriles [72]. This biochemical strategy represents an evolutionary adaptation and a protective mechanism, facilitating the expansion of habitat boundaries [73]. In our experiments, no clear correlation emerged between the concentration of oximes in shoots and the elevation at which plants were growing. However, a high correlation was observed between the increase in nitrile concentrations in both flowers and shoots of *R. linearifolia* and increasing altitude above sea level.

Thus, the metabolic profiles, along with morpho-anatomical data, suggest more favorable developmental conditions for *R. linearifolia* at an altitude of 2687 m a.s.l. compared to 2500 m a.s.l. Furthermore, the increased concentration of secondary metabolite antioxidants observed at 2855 m a.s.l. indicates a heightened environmental stress impact on *R. linearifolia*.

## 4. Materials and Methods

### 4.1. Plant Materials

*R. linearifolia* is a perennial plant characterized by a robust rhizome. The basal leaves are scale-like, gradually increasing in size toward a region just below the inflorescence, then diminishing in size toward the first inflorescence branches. The larger leaves are linear-lanceolate and broadened at the base, ending in a pointed tip. They are either sparsely toothed or entire and sessile. Blooming occurs from May to July, producing a dense, multi-flowered, corymbose inflorescence. The flowers are mostly 5-parted, though less commonly 4-parted. The sepals are greenish, linear, pointed, and about 2.5 times shorter than the petals, which are linear-lanceolate and obtuse and range in color from yellow to brick-red. The stamens, 1.5 times the length of the petals, have red filaments and bright yellow anthers. The fruit is 6–8 mm long with a short beak. 

The collection of *R. linearifolia* samples for experimental purposes at Shymbulak Mountain Resort was conducted on 26 June 2024, during the flowering phase. The sampling sites are part of a single continuous population spread across the entire altitudinal range. It covers almost the entire altitudinal distribution of the species. The characteristics of the sampling sites are presented in Table 3.

Air temperature and relative humidity were measured using Teltonika BLE Multipurpose Sensors (BTSMP1/EYE SENSOR; Vilnius, Lithuania).

### 4.2. Anatomical and Morphological Structure Analysis

Plant material conservation was performed using the Strasburger–Flemming method, initially fixing the specimens in 70% ethanol and subsequently storing them in a 1:1:1 mixture of ethanol, glycerol, and water for preservation [74].

Anatomical sections were prepared using a MZP-01 microtome (Technom, Ekaterinburg, Russia), equipped with a freezing unit OL-ZSO 30 (Inmedprom, Yaroslavl, Russia). with slice thicknesses ranging from 10 to 15 microns. Sections were treated with glycerin and balsam following traditional methods [74]. Micrographs of the sections were captured using a Micro Opix MX 700 (T) microscope (West Medica, Wiener Neudorf, Austria) and a CAM V1200C HD-camera (WestMedica, Wiener Neudorf, Austria) with all anatomical data obtained from 3–5 replicates, analyzing five plants per replicate, using a 40× objective. For each experimental variant, at least five preparations were made. Each parameter was measured in at least three replicates for each preparation.

Plant height was measured in cm under field conditions, and leaf area was determined by the weight method. Paper cutouts of leaf contours were weighed, and leaf area was calculated using the formula:

(1) $$S=\frac{a\times C}{b}$$

where *a* represents the weight of the cutout leaf (mg), *b* represents the weight of a paper square (mg), and *C* represents the area of the paper square (cm^2^).

### 4.3. Photosynthetic Pigment Content Determination

Chlorophylls *a* and *b*, along with carotenoids, were extracted using ethanol and measured post-centrifugation at 4 °C (14,000 rpm). Absorbance readings were taken at wavelengths of 665, 649, and 470 nm using a LEKI SS2107UV spectrophotometer (MEDIORA OY, Helsinki, Finland), following the methods described by Lichtenthaler [75]. All measurements were conducted with a minimum of three biological replicates. All data were obtained from 3 replicates, analyzing 3–5 plants per replicate.

### 4.4. Metabolomic Analysis

The analysis of biologically active organic compounds in *R. linearifolia* was performed using gas chromatography coupled with mass spectrometry (GC-MS) on an Agilent 7890A/5975C system (Agilent Technologies, Santa Clara, CA, USA). Plant tissues (from 3–5 plants per sample) were preserved in 96% ethanol at a ratio of 100 g to 500 mL and extracted twice over 72 h each on an orbital shaker until the ethanol was clear and colorless. Each sample (0.7 µL) was injected into the GC-MS at 310 °C without split flow, with three technical replicates processed for each.

Compounds were separated on a DB-17MS capillary column (60 m × 0.25 mm × 0.25 µm; Agilent Technologies, Santa Clara, CA, USA), with helium as the carrier gas at a flow rate of 1 mL min^−1^. The oven temperature was programmed from 50 to 300 °C at 5 °C min^−1^, with a final 10 min hold at 300 °C. Detection was performed in SCAN *m*/*z* 34–800.

System control and data processing were conducted using Agilent MSD ChemStation software (v. 1701EA, Agilent Technologies, Santa Clara, CA, USA), with retention times, peak areas, and spectral data analyzed. The mass spectra were interpreted using the Wiley 7th edition and NIST 11 libraries, comprising over 550,000 spectra.

### 4.5. Statistical Analysis

The data analysis was conducted using RStudio software (version 2023.06.0 Build 421, RStudio PBC, Boston, MA, USA, 2023). Tukey HSD tests were performed for the pairwise comparisons of the means, while ANOVA was used to confirm statistical significance. Subsequently, the treatments were categorized by letter in descending order, and graphs were generated. Significance was declared at *p* < 0.05.

## 5. Conclusions

The comprehensive analysis of morphophysiological and anatomical parameters, photosynthetic pigments, and the metabolomic spectrum of flowers and shoots of *R. linearifolia* at varying altitudes supports the conclusion that stress on plants intensifies with increased altitude. However, the diverse abiotic factors present in mountainous environments complicate the establishment of a strict linear relationship between altitude and changes in these traits. Nonetheless, among our study sites, the one at 2687 m a.s.l. seems to be the closest to the optimal altitude for the population of studied species in our study system, which, with further research, may be promising for sustainable ecosystem management and the potential introduction of this valuable species. Additionally, the results suggest that the heightened stress at increased altitudes enhances the synthesis of secondary antioxidant metabolites, which could be significant for selecting plants for pharmaceutical applications.

## Figures and Tables

**Table 1:** Changes in anatomical structures (µm) of *R. linearifolia* leaves depending on altitude.

Parameter	Altitude, m a.s.l.				*p*-Value
	2500	2687	2855	3100	
Adaxial epidermis T	31.0 ± 3.58	29.2 ± 1.53	29.0 ± 1.59	26.6 ± 4.58	0.445
Abaxial epidermis T	43.5 ± 2.38 a	41.9 ± 1.70 a	41.6 ± 1.13 a	35.3 ± 3.07 b	<0.01
Central vein T	615 ± 29.1 b	703 ± 53.9 a	629 ± 19.8 ab	567 ± 3.67 b	<0.01
Central vascular bundle D	277 ± 25.1 b	347 ± 26.2 a	216 ± 3.85 c	299 ± 19.1 ab	<0.001
Mesophyll T	319 ± 24.0	314 ± 47.5	296 ± 19.9	312 ± 21.7	0.808
Mesophyll cell D	43.4 ± 3.69	48.4 ± 6.46	43.2 ± 4.51	39.2 ± 2.42	0.181

Note: T—thickness; D—diameter. Different letters within one parameter show significant difference (*p* < 0.05).

**Table 2:** Changes in anatomical structures (µm) of *R. linearifolia* stems depending on altitude.

Parameter	Altitude, m a.s.l.				*p*-Value
	2500	2687	2855	3100	
Epidermis T	31.5 ± 3.56	30.2 ± 4.57	28.0 ± 4.16	26.6 ± 2.73	0.443
Collenchyma T	56.8 ± 2.18 ab	69.3 ± 5.10 a	50.1 ± 9.05 b	50.6 ± 4.54 b	<0.05
Chlorenchyma T	513 ± 4.74 b	608 ± 60.9 a	378 ± 20.1 c	399 ± 25.9 c	<0.001
Conductive bundle D	489 ± 29.3 a	390 ± 32.9 b	268 ± 4.28 c	294 ± 15.4 c	<0.001
Parenchyma T	120 ± 8.98 ab	131 ± 13.9 a	92.3 ± 8.97 c	102 ± 6.60 bc	<0.01
Sclerenchyma T	61.2 ± 4.82	56.5 ± 8.59	55.8 ± 7.74	53.8 ± 8.96	0.696
Endoderm T	55.1 ± 4.56 a	51.8 ± 6.52 a	47.0 ± 3.97 ab	39.0 ± 3.94 b	<0.05
Cell D	115 ± 13.4 a	113 ± 11.4 a	90.8 ± 4.71 ab	85.1 ± 5.13 b	<0.01

Note: T—thickness; D—diameter. Different letters within one parameter show significant difference (*p* < 0.05).

**Table 3:** Characteristics of plant material sampling sites.

Parameter	Unit	Sampling Sites			
		No. 1	No. 2	No. 3	No. 4
Altitude	m a.s.l.	2500	2687	2855	3100
Temperature	°C	28.5	27.3	26.2	24.6
Relative humidity	%	52	52	51	46

## Data Availability

The original contributions presented in the study are included in the article/supplementary material, further inquiries can be directed to the corresponding author.

## Supplementary Materials

The following supporting information can be downloaded at: https://www.mdpi.com/article/10.3390/plants13192698/s1, Table S1. Change in content of SM in flowers of *Rhodiola linearifolia* Boriss. on different altitude above sea level. Table S2. Change in content of SM in shoots of *Rhodiola linearifolia* Boriss. on different altitude above sea level.

## References

[1] Devadoss J., Falco N., Dafflon B., Wu Y., Franklin M., Hermes A., Hinckley E.-L.S., Wainwright H. (2020). Remote Sensing-Informed Zonation for Understanding Snow, Plant and Soil Moisture Dynamics within a Mountain Ecosystem. Remote Sens..

[2] Gao M., Wang X., Meng F., Liu Q., Li X., Zhang Y., Piao S. (2020). Three-Dimensional Change in Temperature Sensitivity of Northern Vegetation Phenology. Glob. Chang. Biol..

[3] Rodríguez-Hernández D. (2019). Secondary Metabolites as a Survival Strategy in Plants of High Mountain Habitats. Boletín Latinoam. Y Del Caribe Plantas Med. Y Aromática.

[4] Li Y., Kong D., Fu Y., Sussman M.R., Wu H. (2020). The Effect of Developmental and Environmental Factors on Secondary Metabolites in Medicinal Plants. Plant Physiol. Biochem..

[5] Terletskaya N.V., Korbozova N.K., Kudrina N.O., Kobylina T.N., Kurmanbayeva M.S., Meduntseva N.D., Tolstikova T.G. (2021). The Influence of Abiotic Stress Factors on the Morphophysiological and Phytochemical Aspects of the Acclimation of the Plant *Rhodiola semenowii* Boriss. Plants.

[6] Terletskaya N.V., Seitimova G.A., Kudrina N.O., Meduntseva N.D., Ashimuly K. (2022). The Reactions of Photosynthetic Capacity and Plant Metabolites of *Sedum hybridum* L. in Response to Mild and Moderate Abiotic Stresses. Plants.

[7] Terletskaya N.V., Shadenova E.A., Litvinenko Y.A., Ashimuly K., Erbay M., Mamirova A., Nazarova I., Meduntseva N.D., Kudrina N.O., Korbozova N.K. (2024). Influence of Cold Stress on Physiological and Phytochemical Characteristics and Secondary Metabolite Accumulation in Microclones of *Juglans regia* L. Int. J. Mol. Sci..

[8] Rahman I.U., Afzal A., Iqbal Z., Hart R., Abd_Allah E.F., Alqarawi A.A., Alsubeie M.S., Calixto E.S., Ijaz F., Ali N. (2020). Response of Plant Physiological Attributes to Altitudinal Gradient: Plant Adaptation to Temperature Variation in the Himalayan Region. Sci. Total Environ..

[9] Pan L., Yang N., Sui Y., Li Y., Zhao W., Zhang L., Mu L., Tang Z. (2023). Altitudinal Variation on Metabolites, Elements, and Antioxidant Activities of Medicinal Plant Asarum. Metabolites.

[10] Veselova L.K., Geldiyeva G.V., Medeu A.R. (2010). Landscapes, Physical-Geographical Zoning.

[11] Ivaschenko A.A. (2008). Flowering Plants of the Southeast of Kazakhstan. Field Guide to the Most Common Species.

[12] Saratikov A.S., Krasnov E.A. (2004). Rhodiola Rosea (Golden Root).

[13] Chaldanbaeva A.K. Biological-Pharmacognostic and Pharmacological Properties of Rhodiola linearifolia of Kyrgyzstan. Proceedings of the 10th International Congress of Phytopharm 2006 “Current Issues of Creating New Medicinal Products of Natural origin”.

[14] Peters K., Worrich A., Weinhold A., Alka O., Balcke G., Birkemeyer C., Bruelheide H., Calf O.W., Dietz S., Dührkop K. (2018). Current Challenges in Plant Eco-Metabolomics. Int. J. Mol. Sci..

[15] Kumari A., Parida A.K. (2018). Metabolomics and Network Analysis Reveal the Potential Metabolites and Biological Pathways Involved in Salinity Tolerance of the Halophyte *Salvadora persica*. Environ. Exp. Bot..

[16] Shih M.-L., Morgan J.A. (2020). Metabolic Flux Analysis of Secondary Metabolism in Plants. Metab. Eng. Commun..

[17] Körner C. (2007). The Use of ‘Altitude’ in Ecological Research. Trends Ecol. Evol..

[18] Ullah R., Khan N., Ali K. (2022). Which Factor Explains the Life-History of *Xanthium strumarium* L., an Aggressive Alien Invasive Plant Species, along Its Altitudinal Gradient?. Plant Direct.

[19] Jahdi R., Arabi M., Bussotti F. (2020). Effect of Environmental Gradients on Leaf Morphological Traits in the Fandoghlo Forest Region (NW Iran). Iforest Biogeosciences For..

[20] Dorogina O.V., Kuban I.N., Achimova A.A., Williams N., Lashchinskiy N.N., Zhmud E.V. (2023). Morphometric Characteristics and Genetic Issr Marker Variability in *Rhodiola rosea* L. (*Crassulaceae*) in Different Ecological and Geographic Conditions in the Altai Republic. Int. J. Mol. Sci..

[21] Cairns D.M. (2013). Alpine Treelines: Functional Ecology of the Global High Elevation Tree Limits. By Christian Körner. Arct. Antarct. Alp. Res..

[22] Tian M., Yu G., He N., Hou J. (2016). Leaf Morphological and Anatomical Traits from Tropical to Temperate Coniferous Forests: Mechanisms and Influencing Factors. Sci. Rep..

[23] Körner C. (1989). The Nutritional Status of Plants from High Altitudes. Oecologia.

[24] Heriyanto, Michalik M., Brotosudarmo T.H.P., Limantara L., Fiedor L. (2014). Reconstitution Approach to Tune Spectral Features of Light Harvesting Complexes for Improved Light Absorption and Energy Transfer. Energy Procedia.

[25] Nevo R., Charuvi D., Tsabari O., Reich Z. (2012). Composition, Architecture and Dynamics of the Photosynthetic Apparatus in Higher Plants. Plant J..

[26] Zielewicz W., Wróbel B., Niedbała G. (2020). Quantification of Chlorophyll and Carotene Pigments Content in Mountain Melick (*Melica nutans* L.) in Relation to Edaphic Variables. Forests.

[27] Li Y., He N., Hou J., Xu L., Liu C., Zhang J., Wang Q., Zhang X., Wu X. (2018). Factors Influencing Leaf Chlorophyll Content in Natural Forests at the Biome Scale. Front. Ecol. Evol..

[28] Lakhanov A.P., Kolomeichenko V.V., Fesenko N.V., Napolova G.V., Muzalevskaya R.S., Savkin V.I., Fesenko A.N. (2004). Morphophysiology and Production Process of Buckwheat.

[29] Rozentsvet O.A., Nesterov V.N., Bogdanova E.S. (2018). The role of the structural organization of the photosynthetic apparatus in the stability of halophytes. Proceedings of the Ecology and Geography of Plants and Plant Communities: Materials of the IV International Scientific Conference.

[30] Ivanov L.A., Migalina S.V., Ronzhina D.A., Tumurjav S., Gundsambuu T., Bazha S.N., Ivanova L.A. (2022). Altitude-Dependent Variation in Leaf Structure and Pigment Content Provides the Performance of a Relict Shrub in Mountains of Mongolia. Ann. Appl. Biol..

[31] Vasileva V., Ilieva A. (2017). Some Physiological Parameters in Mixtures of Cocksfoot and Tall Fescue with Subterranean Clover. Bulg. J. Agric. Sci..

[32] Dymova O.V. (2019). Pigment Complex of Plants in the Taiga Zone of the European Northeast (Organization and Functioning). Doctoral Thesis.

[33] Kurenkova S.V. (1998). Pigment System of Cultivated Plants in the Conditions of the Middle Taiga Subzone of the European North-East.

[34] Santos C.V. (2004). Regulation of Chlorophyll Biosynthesis and Degradation by Salt Stress in Sunflower Leaves. Sci. Hortic..

[35] Mauchamp A., Méthy M. (2004). Submergence-Induced Damage of Photosynthetic Apparatus in *Phragmites australis*. Environ. Exp. Bot..

[36] Eckardt N.A. (2009). A New Chlorophyll Degradation Pathway. Plant Cell.

[37] Li Y., Yang D., Xiang S., Li G. (2013). Different Responses in Leaf Pigments and Leaf Mass per Area to Altitude between Evergreen and Deciduous Woody Species. Aust. J. Bot..

[38] Akram N., Ashraf M. (2011). Improvement in Growth, Chlorophyll Pigments and Photosynthetic Performance in Salt-Stressed Plants of Sunflower (*Helianthus annuus* L.) by Foliar Application of 5-Aminolevulinic Acid. Agrochimica.

[39] Silveira J.A.G., Carvalho F.E.L. (2016). Proteomics, Photosynthesis and Salt Resistance in Crops: An Integrative View. J. Proteom..

[40] Cuttriss A., Pogson B., Davies K.M. (2004). Carotenoids, Plant Pigments and Their Manipulation. Carotenoids Chapter: Plant Pigments and Their Manipulation.

[41] Demmig-Adams B., Gilmore A.M., Iii W.W.A. (1996). In Vivo Functions of Carotenoids in Higher Plants. FASEB J..

[42] Zheng W., Yu S., Zhang W., Zhang S., Fu J., Ying H., Pingcuo G., Liu S., Zhao F., Wu Q. (2023). The Content and Diversity of Carotenoids Associated with High-Altitude Adaptation in Tibetan Peach Fruit. Food Chem..

[43] Maslova T., Popova I. (1993). Adaptive Properties of the Plant Pigment Systems. Photosynthetica.

[44] Llorente B., Torres-Montilla S., Morelli L., Florez-Sarasa I., Matus J.T., Ezquerro M., D’Andrea L., Houhou F., Majer E., Picó B. (2020). Synthetic Conversion of Leaf Chloroplasts into Carotenoid-Rich Plastids Reveals Mechanistic Basis of Natural Chromoplast Development. Proc. Natl. Acad. Sci. USA.

[45] Sun T., Rao S., Zhou X., Li L. (2022). Plant Carotenoids: Recent Advances and Future Perspectives. Mol. Hortic..

[46] Torres-Montilla S., Rodriguez-Concepcion M. (2021). Making Extra Room for Carotenoids in Plant Cells: New Opportunities for Biofortification. Prog. Lipid Res..

[47] Wang Y., Zhang C., Dong B., Fu J., Hu S., Zhao H. (2018). Carotenoid Accumulation and Its Contribution to Flower Coloration of *Osmanthus fragrans*. Front. Plant Sci..

[48] Terletskaya N.V., Korbozova N.K., Grazhdannikov A.E., Seitimova G.A., Meduntseva N.D., Kudrina N.O. (2022). Accumulation of Secondary Metabolites of *Rhodiola semenovii* Boriss. In Situ in the Dynamics of Growth and Development. Metabolites.

[49] Marak H.B., Biere A., Damme J.M.M.V. (2003). Fitness Costs of Chemical Defense in Plantago Lanceolata l.: Effects of Nutrient and Competition Stress. Evolution.

[50] Rasmann S., Pellissier L., Defossez E., Jactel H., Kunstler G. (2014). Climate-Driven Change in Plant–Insect Interactions along Elevation Gradients. Funct. Ecol..

[51] Körner C., Körner C. (2021). Alpine Treelines. Alpine Plant Life: Functional Plant Ecology of High Mountain Ecosystems.

[52] He M., He C.-Q., Ding N.-Z. (2018). Abiotic Stresses: General Defenses of Land Plants and Chances for Engineering Multistress Tolerance. Front. Plant Sci..

[53] Boncan D.A.T., Tsang S.S.K., Li C., Lee I.H.T., Lam H.-M., Chan T.-F., Hui J.H.L. (2020). Terpenes and Terpenoids in Plants: Interactions with Environment and Insects. Int. J. Mol. Sci..

[54] Yuldasheva N.K., Gusakova S.D., Nurullaeva D.K., Farmanova N.T., Zakirova R.P., Kurbanova E.R. (2020). Neutral Lipids of Oats Fruit (*Avena sativa* L.). Drug Dev. Regist..

[55] Lee J., Moraes-Vieira P.M., Castoldi A., Aryal P., Yee E.U., Vickers C., Parnas O., Donaldson C.J., Saghatelian A., Kahn B.B. (2016). Branched Fatty Acid Esters of Hydroxy Fatty Acids (FAHFAs) Protect against Colitis by Regulating Gut Innate and Adaptive Immune Responses. J. Biol. Chem..

[56] Martinière A., Gayral P., Hawes C., Runions J. (2011). Building Bridges: Formin1 of Arabidopsis Forms a Connection between the Cell Wall and the Actin Cytoskeleton. Plant J..

[57] Sui N., Han G. (2014). Salt-Induced Photoinhibition of PSII Is Alleviated in Halophyte *Thellungiella halophila* by Increases of Unsaturated Fatty Acids in Membrane Lipids. Acta Physiol. Plant.

[58] Liu S., Wang W., Li M., Wan S., Sui N. (2017). Antioxidants and Unsaturated Fatty Acids Are Involved in Salt Tolerance in Peanut. Acta Physiol. Plant.

[59] Polturak G., Heinig U., Grossman N., Battat M., Leshkowitz D., Malitsky S., Rogachev I., Aharoni A. (2018). Transcriptome and Metabolic Profiling Provides Insights into Betalain Biosynthesis and Evolution in *Mirabilis jalapa*. Mol. Plant.

[60] Cara C., Gauvin-Lepage J., Lefebvre H., Létourneau D., Alderson M., Larue C., Beauchamp J., Gagnon L., Casimir M., Girard F. (2016). Le Modèle humaniste des soins infirmiers -UdeM: Perspective novatrice et pragmatique. Rech. En Soins Infirm..

[61] Farmer E.E. (2001). Surface-to-Air Signals. Nature.

[62] Sharkey T.D., Yeh S. (2001). Isoprene Emission from Plants. Annu. Rev. Plant Biol..

[63] De Jersey J., Zerner B. (1969). Spontaneous and Enzyme-Catalyzed Hydrolysis of Saturated Oxazolinones. Biochemistry.

[64] Aryan Y. (2005). 5-Substituted Furfurylidene (Benzylidene)-1,3-Oxazol-4-Ones: Synthesis, Stereostructure, Reactions and Biological Action. Ph.D. Thesis.

[65] Alonso-Amelot M.E., Usubillaga A., Ávila-Núñez J.L., Oliveros-Bastidas A., Avendaño M. (2006). Effects of Minthostachys Mollis Essential Oil and Volatiles on Seedlings of Lettuce, Tomato, Cucumber and *Bidens pilosa*. Allelopath. J..

[66] Camas N., Radusiene J., Ivanauskas L., Jakstas V., Cirak C. (2014). Altitudinal Changes in the Content of Bioactive Substances in *Hypericum orientale* and *Hypericum pallens*. Acta Physiol. Plant.

[67] Padilla-González G.F., Diazgranados M., Da Costa F.B. (2017). Biogeography Shaped the Metabolome of the Genus *Espeletia*: A Phytochemical Perspective on an Andean Adaptive Radiation. Sci. Rep..

[68] Nasri Z., Ahmadi M., Striesow J., Ravandeh M., von Woedtke T., Wende K. (2022). Insight into the Impact of Oxidative Stress on the Barrier Properties of Lipid Bilayer Models. Int. J. Mol. Sci..

[69] Hashim A.M., Alharbi B.M., Abdulmajeed A.M., Elkelish A., Hozzein W.N., Hassan H.M. (2020). Oxidative Stress Responses of Some Endemic Plants to High Altitudes by Intensifying Antioxidants and Secondary Metabolites Content. Plants.

[70] Toderich K.N., Terletskaya N.V., Zorbekova A.N., Saidova L.T., Ashimuly K., Mamirova A., Shuyskaya E.V. (2023). Abiotic Stresses Utilisation for Altering the Natural Antioxidant Biosynthesis in *Chenopodium quinoa* L. Russ. J. Plant Physiol..

[71] Gleadow R.M., Møller B.L. (2014). Cyanogenic Glycosides: Synthesis, Physiology, and Phenotypic Plasticity. Annu. Rev. Plant Biol..

[72] Sibbesen O., Koch B.M., Rouze P., Moller B.L., Halkier B.A., Wallsgrove R.M. (1995). Biosynthesis of Cyanogenic Glucosides. Elucidation of the Pathway and Characterization of the Cytochromes P-450 Involved in Amino Acids and Their Derivatives in Higher Plants.

[73] Lesovskaya M. (2020). Antioxidant Activity of Cyanogenic Vegetable Raw Materials.

[74] Barykina R., Veselova T., Devyatov A., Dzhalilova K.K., Ilyina G., Chubatova N. (2004). Guide on Botanical Microtechique. Basics and Methods.

[75] Lichtenthaler H.K. (1987). [34] Chlorophylls and Carotenoids: Pigments of Photosynthetic Biomembranes. Methods in Enzymology.

