# Supplementary material for: Altitude-Dependent Morphophysiological, Anatomical, and Metabolomic Adaptations in Rhodiola linearifolia Boriss
Source: Plants (Basel). 2024 Sep 26;13(19):2698. doi: 10.3390/plants13192698 (PMC11479101; doi:10.3390/plants13192698)
Supplement: Supplementary file 1 [file plants-13-02698-s001.zip › plants-3205769-supplementary.pdf]

Table S1. Change in content of SM in flowers of *Rhodiola linearifolia* Boriss. on different altitude above sea level

	1 (43°07'25"N 77°05'27"E 2500м)			2 (43°07'16"N 77°05'49"E 2687м)			3 (43°07'08"N 77°06'10"E 2855 м)			4 (43°06'57"N 77°06'37"E 3100 м)		
	Content, %	Retention time, min	Identification probability, %	Content, %	Retention time, min	Identification probability, %	Content, %	Retention time, min	Identification probability, %	Content, %	Retention time, min	Identification probability, %
5(4H)-oxazolone derivatives												
5(4H)-Oxazolone, 4,4'-(1,4-phenylenedimethylidyne)bis [2-phenyl-	-	-	-				29.65	6.55	70			
sum							29.65					
saturated monocyclic hydrocarbons												
Cyclotetradecane	1.03	27.45	79									
sum	1.03											
fatty acid and fatty acid esters												
Tetradecanoic acid				0.17	29.56	72						
Hexadecanoic acid				20.95	33.71	88						
Octadecanoic acid				15.36	37.44	92						
Propanoic acid, 2-methyl-, 2-methylbutyl ester												
Hexadecanoic acid, ethyl ester (palmitic acid ester)							3.26	38.72	89	6.09	38.73	91
Ethyl Oleate (oleic acid ester)							--	-	-	1.03	42.43	78
Ethyl 9,12,15-octadecatrienoate (Ethyl 9α-linolenate, linolenic acid ethyl ester)												
Octadecanoic acid	5.33	37.43	90									
Oleic Acid	1.88	37.52	73									
9,12-Octadecadienoic acid, ethyl ester (linoleic acid ester)	5.21	37.75	91	5.54	37.75	91	6.58	42.76	85	8.24	42.76	91

9,12-Octadecadienoic acid (Z,Z)-	1.42	37.88	77	2.02	37.89	73						
9,12,15-Octadecatrienoic acid, ethyl ester, (Z,Z,Z)-	11.12	38.28	94	8.01	38.28	94	6.37	43.29	90	14.09	43.30	93
9,12,15-Octadecatrienoic acid, (Z,Z,Z)-	1.04	38.42	82	1.18	38.42	74						
Hexanedioic acid, bis(2-ethylhexyl) ester	1.10	41.83	87	1.61	41.83	91						
sum	27.1			54.39			16.36			29.39		
nitriles												
3-Butenenitrile, 2-methyl-				-	-	-	-	-	-	5.82	7.73	74
Butanenitrile, 2-methylene-				-	-	-	1.32	9.55	71	-	-	-
Propanenitrile, 3-methoxy-	1.97	10.92	62	0.94	10.84	67	1.28	15.20	68	1.48	15.08	67
Propanedinitrile, (phenylmethylene)-	0.50	27.94	77	0.36	27.94	79						
sum	2.47			1.30			2.60			7.30		
oximes												
Oxime-, methoxy-phenyl-	0.98	7.56	79				5.85	10.97	78	1.88	10.69	79
sum	0.98						5.85			1.88		
alcohols and their derivatives												
(S)-(+)-1,2-Propanediol				0.42	6.36	92						
1-Propanol, 2-(2-hydroxypropoxy)-	1.46	13.28	82	0.96	13.24	76						
Ethanol, 2,2'-oxybis-	0.32	12.09	79				-	-	-	0.48	16.50	71
Glycerin				0.85	12.65	74	-	-	-	1.39	17.14	68
Cyclopropyl carbinol	0.76	18.23	70	0.39	18.21	65						
2-Propanol, 1,1'-[(1-methyl-1,2-ethanediyl)bis(oxy)]bis-	0.96	21.15	77	0.69	21.14	80						
1-Dodecanol	0.88	22.72	91	1.07	22.72	92						
α-Methyl-α-[4-methyl-3-pentenyl]oxiranemethanol	2.82	23.51	82									
Propanol, [(butoxymethylethoxy)methylethoxy]-	0.57	28.47	75	0.94	28.47	95						
Ethanol, 2-(dodecyloxy)-	1.40	28.76	79	1.88	28.75	90						
Ethanol, 2-(tetradecyloxy)-	1.12	32.97	86	1.37	32.97	86						
Propylene Glycol				0.47	6.54	92						

Diethylene glycol monododecyl ether				2.00	35.32	87						
Triethylene glycol monododecyl ether	1.81	40.96	86	1.90	40.96	87						
Tetraethylene glycol monododecyl ether	2.22	48.78	74	1.25	45.91	80						
Hexaethylene glycol monododecyl ether				2.08	48.78	72						
Pentaethylene glycol monododecyl ether	0.93	50.62	65									
sum	15.25			16.27						1.87		
aldehydes and their derivatives												
Benzeneacetaldehyde	1.65	14.69	91				0.93	19.40	88	0.91	19.39	88
5-Hydroxymethylfurfural	0.61	21.61	68	0.59	21.61	73						
sum	2.26			0.59			0.93			0.91		
ketones and their derivatives												
2-Propanone, 1-hydroxy-							9.99	8.21	91	13.91	7.60	94
1-Cyclohexanone, 2-methyl-2-(3-methyl-2-oxobutyl)	6.42	44.44	65									
4-Cyclopentene-1,3-dione	0.90	10.49	66	1.06	10.46	72						
1,2-Cyclopentanedione	1.53	11.21	93	1.47	11.13	97				1.74	15.35	87
1,2-Cyclopentanedione, 3-methyl-							-	-	-	1.95	18.52	83
2-Cyclopenten-1-one, 2-hydroxy-3-methyl-	0.61	13.93	83	0.40	13.89	79	2.20	18.57	66	-	-	-
4-Penten-2-one, 3-methyl-				0.44	15.12	72						
sum	9.46			3.37			12.19			17.60		
esters and their derivatives												
Benzoic acid, 4-ethoxy-, ethyl ester	0.36	27.39	71									
1,2-Ethanediol, monoacetate	0.75	7.43	80	0.81	7.26	82	-	-	-	1.52	10.36	80
3-Methyl-2-butenoic acid, cyclobutyl ester				1,88	18.66	76						
2-Hydroxy-gamma-butyrolactone	0.91	14.95	86	0.62	14.91	82	0.78	19.66	74	1.86	19.63	83
Butyrolactone							0.79	17.17	73	0.75	17.09	72
sum	2.02			3.31			1.57			4.13		

amides												
Urea, 1-methylcyclopropyl-	1.78	11.46	71									
sum	1.78											
dioxepine derivatives												
5H-1,4-Dioxepin, 2,3-dihydro-7-methyl-	1.43	14.45	67	1.93	14.14	67						
sum	1.43			1.93								
1,4-dioxin derivatives												
1,4-Dioxin, 2,3-dihydro-5,6-dimethyl-	0.40	15.15	73									
sum	0.40											
furan and pyran derivatives												
2-Furanmethanol				1.20	8.66	70						
2-Furancarboxaldehyde, 5-methyl-				0.86	12.20	70						
2(5H)-Furanone heterocyclic compounds butenolide?	0.60	12.84	84	0.73	12.78	80	-	-	-	0.91	17.29	85
Ethyl 2-(5-methyl-5-vinyltetrahydrofuran-2-yl)propan-2-yl carbonate	13.61	13.12	92	4.05	13.12	90	7.11	17.69	91	5.25	17.68	82
2,5-Dimethyl-4-hydroxy-3(2H)-furanone	0.87	14.35	79	0.57	14.31	75	-	-	-	0.65	20.94	71
2H-Pyran-3-ol, 6-ethenyltetrahydro-2,2,6-trimethyl-	4.48	16.38	91	3.81	16.37	90	4.12	21.22	88	2.93	21.20	78
4H-Pyran-4-one, 2,3-dihydro-3,5-dihydroxy-6-methyl-	1.63	17.46	79	0.52	17.45	73						
2-Furanmethanol, 5-ethenyltetrahydro-α,α,5-trimethyl-, cis-				3.01	23.58	79						
Benzofuran, 2,3-dihydro-							-	-	-	0.40	24.26	74
α-d-Lyxofuranoside, methyl	0.56	26.51	67									
Ethyl α-d-glucopyranoside	5.35	31.51	85									

β-D-Glucopyranose, 1,6-anhydro-							-	-	-	0.59	34.63	68
α-d-Glucofuranosyl benzenesulfonate	3.24	45.05	64									
sum	30,34			14.75			11.23			10.73		
pyrimidine derivatives												
2,4,5-Trihydroxypyrimidine				0.28	16.14	71						
sum				0.28								
phenolic compounds												
2,2-Diethoxyacetophenone							2.28	14.53	65	1.80	14.53	66
Phenol	0.78	11.82	85	0.63	11.78	77	1.52	16.18	87	1.69	16.12	90
Phenol, 3-methyl-							1.15	19.21	67	1.40	19.20	67
Catechol	1.26	18.82	91				-	-	-	1.31	23.67	86
Phenol, 2-(1,1-dimethylethyl)-4-methyl-							1.59	26.71	91	1.34	26.71	88
Phenol, 2-(1,1-dimethylethyl)-6-methyl-										1.06	26.71	88
2-Methoxy-4-vinylphenol	0.84	22.25	86	0.59	22.24	86				0.72	27.22	77
4,4'-(Hexafluoroisopropylidene) diphenol	0.57	39.54	85	1.09	39.54	87						
Phenol, 2,2'-methylenebis[6-(1,1-dimethylethyl)-4-methyl-							16.44	50.64	92	15.46	50.64	91
sum	2.88			2.31			22.98			24.78		
terpenes												
Bicyclo[4.1.0]heptan-3-one, 4,7,7-trimethyl-, [1R-(1*α*,4*α*,6*α*)]-				-	-	-	-	-	-	0.91	21.12	66
Phytol, acetate				-	-	-	-	-	-	-	-	-
Phytol	1.11	36.10	79	1.5	36.10	83-	1.49	41.11	88	1.47	41.11	80
3,7,11,15-Tetramethyl-2-hexadecen-1-ol				-	-	-	1.15	34.12	77	0.91	34.13	81
6-*O*-Acetyl-1-[[4-bromophenyl]thio]-*β-D*-glucoside S,S-dioxide	2.47	46.51	65									
sum	3.58			1.5			2.64			3.29		

Table S2. Change in content of SM in shoots of *Rhodiola linearifolia* Boriss. on different altitude above sea level

	1			2			3			4		
	Content, %	Retention time, min	Identification probability, %	Content, %	Retention time, min	Identification probability, %	Content, %	Retention time, min	Identification probability, %	Content, %	Retention time, min	Identification probability, %
fatty acid and fatty acid esters												
3-Methyl-2-butenoic acid, 2-chlorophenyl ester				1.21	18.66	68						
Hexadecanoic acid	2.92	33.66	82	1.27	33.66	69						
Hexadecanoic acid, ethyl ester (palmitic acid ester)							2.05	38.73	88	3.24	38.73	91
Octadecanoic acid				0.71	37.41	75						
9,12-Octadecadienoic acid, ethyl ester (linoleic acid ester)	0.30	37.75	75	1.73	37.75	88	1.47	42.76	85	2.78	42.76	90
9,12-Octadecadienoic acid (Z,Z)-	0.61	37.87	74	0.63	37.87	69						
9,12,15-Octadecatrienoic acid, (Z,Z,Z)-	0.99	38.41	85	0.76	38.41	70						
9,12,15-Octadecatrienoic acid, ethyl ester, (Z,Z,Z)-				7.34	38.28	93	7.38	43.30	93	7.62	43.29	93
Ethyl 9,12,15-octadecatrienoate	1.49	38.28	90									
Hexanedioic acid, bis(2-ethylhexyl) ester	0.61	41.83	84	1.33	41.83	83						
sum	6.92			14.98			10.9			13.64		
nitriles												
2-Pentenenitrile				8.30	6.87	77						
3-Butenenitrile, 2-methyl-							7.01	7.74	78	8.58	7.76	89
2-Methyl-2-butenenitrile							4.61	9.52	84	-	-	-
Butanenitrile, 2-methylene-							-	-	-	5.47	9.55	83
Propanenitrile, 3-methoxy-	3.56.	11.02	70	4.07	10.94	69	2.42	15.19	65	-	-	-
sum	3.56			4.07			14.0			14.05		

oximes												
Oxime-, methoxy-phenyl-_	5.04	7.73	80	1.43	7.60	83	3.86	10.92	81	4.15	10.92	79
sum	5.40			13.80			3.86			4.15		
carbohydrates and their derivatives												
D-Allose	1.20	29.65	74									
1,4:3,6-Dianhydro-*α*-d-glucopyranose				0.44	21.22	71						
α-d-Lyxofuranoside, methyl	2.01	26.52	73									
β-D-Glucopyranose, 1,6-anhydro-				1.00	29.63	77	1.99	34.62	77	1.12	34.63	86
Ethyl *α-D*-glucopyranoside	3.71	32.97	77	6.18	31.48	85				0.39	36.48	74
β-D-Glucopyranose, 4-O-β-D-galactopyranosyl-				0.57	33,60	63						
α-Methyl-D-mannopyranoside	3.89	42.69	69									
sum	10.81			8.19			1.99			1.51		
alcohols and their derivatives												
R-(-)-1,2-propanediol	1.64	6.80	83									
Ethanol, 2,2'-oxybis-	0.69	12.16	90	1.00	12.10	88						
1-Propanol, 2,2'-oxybis-	0.49	13.42	88	0.94	13.30	90						
1-Propanol, 2-(2-hydroxypropoxy)-	0.46	13.34	87	0.95	13.39	88						
Glycerin							-	-	-	1.31	17.12	77
Cyclopropyl carbinol	2.00	18.25	74	1.41	18.23	71						
2-Propanol, 1,1'-[(1-methyl-1,2-ethanediyl)bis(oxy)]bis-	0.93	21.16	80	0.87	21.15	83						
Phenylethyl Alcohol							0.66	21.36	81			
1-Dodecanol	0.56	22.72	88									
Triethylene glycol				1.36	22.72	92	-	-	-	0.20	25.39	77
4,7,10,13,16-Pentaoxanonadeca-1,18-diene				0.42	23.75	66						
2,5,8,11,14-Pentaoxahexadecan-16-ol	0.73	28.33	75									
Propanol, [(butoxymethylethoxy)methylethoxy]-				1.01	28.47	73						

Tri(propylene glycol) propyl ether	-	-	-	0.70	28.53	64	-	-	-	-	-	-
Ethanol, 2-(dodecyloxy)-	1.13	28.76	84	2.36	28.76	88	-	-	-	-	-	-
Ethanol, 2-(tetradecyloxy)-	3.61	32.97	77	2.20	32.97	85	-	-	-	-	-	-
-1-Hexadecanol	-	-	-	1.35	27.45	86	-	-	-	-	-	-
Propylene Glycol	-	-	-	0.72	6.62	86	-	-	-	-	-	-
Triethylene glycol	0.90	20.45	82	0.61	20.43	74	-	-	-	-	-	-
Diethylene glycol monododecyl ether	1.32	35.32	84	-	-	-	-	-	-	-	-	-
Triethylene glycol monododecyl ether	1.56	40.95	85	2.76	40.95	83	-	-	-	-	-	-
Tetraethylene glycol monododecyl ether	1.01	45.91	75	2.06	45.91	70	-	-				
Heptaethylene glycol monododecyl ether	0.81	50.62	66	2.02	48.78	66						
Pentaethylene glycol monododecyl ether	-	-	-	0.99	50.62	65						
sum	17.84			23.12			0.66			1.51		
aldehydes and their derivatives												
Benzeneacetaldehyde	1.71	14.70	90	0.82	14.69	86	0.31	19.40	68	-	-	-
sum	1.71			0.82			0.31					
ketones and their derivatives												
2-Propanone, 1-hydroxy-	11.47	6.23	90				13.36	8.10	92	9.36	8.11	93
5-Hexen-2-one				0.99	15.15	68						
4-Cyclopentene-1,3-dione	1.05	10.52	63	-	-	-	-	-	-	1.01	14.55	65
2-Cyclopenten-1-one, 2-hydroxy-							-	-	-	1.08	15.45	90
1,2-Cyclopentanedione	2.68	11.30	94	2.65	11.22	92	1.01	15.46	80	-	-	-
2-Cyclopenten-1-one, 2-hydroxy-3-methyl-	1.28	13.98	89	0.90	13.94	87	-	-	-	-	-	-
1,2-Cyclopentanedione, 3-methyl-							1,71	18.57	75	1.25	18.57	90
4-Cyclopentene-1,3-dione, 4 methoxy-5-methyl-	1.16	18.69	70									
2-Cyclopenten-1-one, 3-ethyl-2-hydroxy-							0.32	21.14	70	-	-	-
sum	17.64			4.54			16.40			12.70		

carboxylic acid and their derivatives												
Acetic anhydride				-	-	-	-	-	-	1.44	6.18	85
Acetic acid				-	-	-	4.28	6.42	94	0.77	6.44	94
Butanoic acid, 4-hydroxy-	3.61	12.73	85	0.57	12.60	81	0.67	17.16	86			
1,2-Ethanediol, monoacetate	2.13	7.63	83				-	-	-	1.28	10.65	78
Methyl 2,5,8,11,14-pentaoxahexadecan-16-oate				0.75	28.33	68						
(*S*)-(+)-2',3'-Dideoxyribonolactone	0.93	21.63	85				-	-	-	0.52	26.59	75
Urea, 1-methylcyclopropyl-	2.34	11.55	70	2.53	11.48	71						
sum	9.01			3.79			4.95			4,01		
amino acid and their derivatives												
l-Alanine, N-(2-furoyl)-, propyl ester				0.31	22.12	73						
l-Alanine, N-(2-furoyl)-, ethyl ester	0.52	21.12	75									
sum	0.52			0.31								
dioxolanone derivative												
1,3-Dioxol-2-one,4,5-dimethyl-	4.24	14.57	70	1.77	14.56	71						
sum	4.24			1.77								
furan and pyran derivatives												
2-Furanmethanol	0.51	11.76	74									
Butyrolactone				2.58	12.68	84	-	-	-	-	-	-
2(5H)-Furanone	1.85	12.90	89	1.32	12.85	87	1.63	17.36	84	0,8	17.35	93
--2-Hydroxy-gamma-butyrolactone	3.84	15.03	85	2.33	14.96	88	2.48	19.66	82	2.02	19.68	88
2-Furanmethanol, 5-ethenyltetrahydro-α,α,5-trimethyl-, cis-				1.40	16.35	68						
4H-Pyran-4-one, 2,3-dihydro-3,5-dihydroxy-6-methyl-	1.76	17.49	82	0.79	17.47	65						
2(3H)-Furanone, 5-acetyldihydro-	0.45	19.56	88	0.43	19.55	77						
4H-Pyran-4-one, 3-hydroxy-2,6-dimethyl-	0.94	19.87	78	0.35	19.83	73						

1,2-Ethanediol, 1-(2-furanyl)-	1.61	20.71	66									
2H-Pyran-3-ol, 6-ethenyltetrahydro-2,2,6-trimethyl-							-	-	-	0.68	21.22	77
Benzofuran, 2,3-dihydro-	0.76	19.36	77				-	-	-	0.35	24.26	76
sum	11.73			9.20			4.11			3.85		
phenolic compounds												
Phenol				1.14	11.84	79	0.90	16.17	77	1.08	16.15	81
Phenol, 3-methyl-							0.37	19.21	66			
Catechol	1.69	18.85	92	0.98	18.81	80				1.75	23.69	90
Benzene, 1,4-dimethoxy-	0.61	19.28	80	0.37	19.28	73						
Benzoic acid, 4-ethoxy-, ethyl ester				0.47	27.39	69						
Phenol, 2-(1,1-dimethylethyl)-3-methyl-							0.73	26.71	87	-	-	-
Phenol, 2-(1,1-dimethylethyl)-4-methyl-							-	-	-	11.48	26.71	90
Phenol, 2-(1,1-dimethylethyl)-5-methyl-							-	-	-			
2-Methoxy-4-vinylphenol	0.86	22.25	86	0.80	22.45	80	0.42	27.22	75	0.55	27.22	75
4,4'-(Hexafluoroisopropylidene) diphenol	0.25	39.54	76	0.50	39.54	80						
Phenol, 2,2'-methylenebis[6-(1,1-dimethylethyl)-4-methyl-							16.00	50.64	92	19.05	50.64	92
Ethanone, 2-(formyloxy)-1-phenyl-							15.59	6.48	74			
sum	3.41			4.26			34.01			33.91		
terpenes												
α-Methyl-α-[4-methyl-3-pentenyl]oxiranemethanol							-	-	-	2.04	1769	85
Phytol, acetate							2.34	34.13	85	-	-	-
Isophytol							0.33	37.13	71	-	-	-
Phytol	6.52	36.10	89	11.15	36.10	94	5.41	41.11	88	7.77	41.11	93
3,7,11,15-Tetramethyl-2-hexadecen-1-ol							0.73	35.20	78	1,86	34.13	88

sum	6.52			11.15			8.81			11.67		
